# Stevens Triennial Prize

**Published:** 1873-06

**Authors:** J. C. Dalton


### Stevens Triennial Prize.

The Stevens Triennial Prize for 1873 has been awarded to an Essay on "The Sphygmograph; its Physiological and Pathological Indications," by Edgar Holden, M. D., of Newark, N. J. The questions proposed for the next prize (1876) are as follows:

I. "The History of Epidemic Diseases in the United States, from 1860 to 1870;" statements as to localities, dates, extent of prevalence, and mortality, to be authenticated by appropriate references. The question of *treatment* is not to form a part of the above subject.

II. "The use of the Spectroscope in its application to Scientific and Practical Medicine."

The competing essays on either of the above subjects must be sent in to the President of the College of Physicians and Surgeons, New York, on or before the first day of January, 1876. Each essay must be designated by a device or motto, must be accompanied by a sealed envelope, bearing the same device or motto and containing the name and address of the author. The envelope belonging to the successful essay will be opened, and the name of the author announced, at the Annual commencement of the College, in March, 1876.

This Prize, which will amount to two hundred dollars is open for universal competition. Per order of the Prize Committee. J. C. DALTON, M. D.

Obituary Notice.—We have the sad duty of announcing the death of Dr. D. V. Stranahan, of Warren, Pa., at his home on the 19th of May. We await further particulars.

